# Saliva changes in composition associated to COVID-19: a preliminary study

**DOI:** 10.1038/s41598-022-14830-6

**Published:** 2022-06-27

**Authors:** Alberto Muñoz-Prieto, Ivana Rubić, Juan Carlos Gonzalez-Sanchez, Josipa Kuleš, Silvia Martínez-Subiela, José Joaquín Cerón, Enrique Bernal, Alberto Torres-Cantero, María Rosario Vicente-Romero, Vladimir Mrljak, Asta Tvarijonaviciute

**Affiliations:** 1 Clinic for Internal Diseases. Faculty of Veterinary Medicine, University of Zagreb, Heinzelova 55, 10000 Zagreb, Croatia; 2 Interdisciplinary Laboratory of Clinical Analysis, Regional Campus of International Excellence ‘Campus Mare Nostrum’, Interlab-UMU, University of Murcia, 30100 Murcia, Spain; 3 BioQuant, Heidelberg University, Im Neuenheimer Feld 267, Heidelberg, Germany; 4 Unit of Infectious Diseases, Hospital General Universitario Reina Sofía, Universidad de Murcia, 30003 Murcia, Spain; 5 Preventive Medicine, Hospital Clínico Universitario Virgen de La Arrixaca, IMIB, Universidad de Murcia, 30120 Murcia, Spain; 6 Unit of Microbiology, Hospital General Universitario Reina Sofía, Universidad de Murcia, 30003 Murcia, Spain

**Keywords:** Proteomics, Infectious diseases

## Abstract

The coronavirus disease 2019 (COVID-19), caused by the severe acute respiratory syndrome coronavirus 2 (SARS-CoV 2), is usually associated with a wide variety of clinical presentations from asymptomatic to severe cases. The use of saliva as a diagnostic and monitoring fluid has gained importance since it can be used to investigate the immune response and to direct quantification of antibodies against COVID-19. Additionally, the use of proteomics in saliva has allowed to increase  our understanding of the underlying pathophysiology of diseases, bringing new perspectives on diagnostics, monitoring, and treatment. In this work, we compared the salivary proteome of 10 patients with COVID-19, (five patients with mild and five patients with severe COVID-19) and ten control healthy patients. Through the application of proteomics, we have identified 30 proteins whose abundance levels differed between the COVID-19 groups and the control group. Two of these proteins (TGM3 and carbonic anhydrase-CA6) were validated by the measurement of gGT and TEA respectively, in 98 additional saliva samples separated into two groups: (1) COVID-19 group, integrated by 66 patients who tested positive for COVID-19 (2) control group, composed of 32 healthy individuals who did not show any sign of disease for at least four weeks and were negative for COVID-19 in RT-PCR. In the proteomic study there were observed upregulations in CAZA1, ACTN4, and ANXA4, which are proteins related to the protective response against the virus disturbance, and the upregulation of TGM3, that is correlated to the oxidative damage in pulmonary tissue. We also showed the downregulation in cystatins and CA6 that can be involved in the sensory response to stimulus and possibly related to the presence of anosmia and dysgeusia during the COVID-19. Additionally, the presence of FGB in patients with severe COVID-19 but not in mild COVID-19 patients could indicate a higher viral aggregation and activation in these cases. In conclusion, the salivary proteome in patients with COVID-19 showed changes in proteins related to the protective response to viral infection, and the altered sensory taste perception that occur during the disease. Moreover, gGT and TEA could be potential biomarkers of respiratory complications that can occurs during COVID 19 although further larger studies should be made to corroborate this.

## Introduction

The severe acute respiratory syndrome coronavirus 2 (SARS-CoV-2) has been spreading worldwide since at least December 2019, causing the coronavirus disease 2019 (COVID-19)^1^. The most common clinical signs of this disease are the development of respiratory distress syndrome, loss of sensory acuity affecting both smell and taste, sore throat, fever, muscle weakness, tiredness, headache, and diarrhea^2^.

Saliva has gained special importance in recent years as a diagnostic fluid since it is easy to obtain by a non-invasive sampling process. The SARS-CoV-2 virus can be detected in this fluid as well as the immunoglobulins directly produced after the immune response^3–5^. Overall, saliva could serve as a possible source of potential biomarkers in the clinical management of patients with COVID-19 ^5,6^.

Proteomic studies allow the sensitive analysis of proteins at a larger scale to identify those that can be related to different physiological and metabolic states, as well as to help elucidate their biological role. Moreover, differential expression analyses can detect proteins that are either over-or under-expressed in a given disease process and thus may serve as biomarkers of the disease^7^. Proteomics, therefore, can contribute to a better understanding of the underlying pathophysiology and potentially serve as a diagnostic and monitoring tool, as well as opening new treatment perspectives. The application of labeling techniques (tandem mass tag, TMT) to peptide quantification has increased the number of identified proteins in proteomics analysis, with a considerable increment of the technique’s sensitivity^8^.

The use of saliva in proteomics has been applied in a large spectrum of human diseases^9^ such as periodontitis^10^, oral cancer^11^, autoimmune diseases like ﻿Sjogren’s syndrome^12^, burning mouth syndrome^13^, and infectious diseases like Zika virus^14^. However, to the authors’ knowledge, there are no published studies about changes in the proteome of saliva in patients with COVID-19 .

The  hypothesis of this study was that Covid-19 can produce changes in saliva composition that could be detected by proteomics. Therefore, the objective of this report was to perform a proteomic study of the saliva of SARS-CoV-2 patients. A deeper knowledge of potential changes in the saliva proteome can improve our understanding of the mechanisms associated and identify new possible biomarkers for this disease.

## Results

### Salivary proteomic profile in COVID-19

The high-resolution proteomic analysis allowed the identification of 537 proteins from the 20 saliva samples (Data is available via ProteomeXchange Consortium with identifier PXD031318). The univariate analysis comparing the two groups (COVID-19 versus control) revealed 30 proteins showing significant differences in abundance between them, where 20 proteins were downregulated and ten were upregulated in the saliva of patients with COVID-19 (Supplementary Table S1).

**Figure 1:**
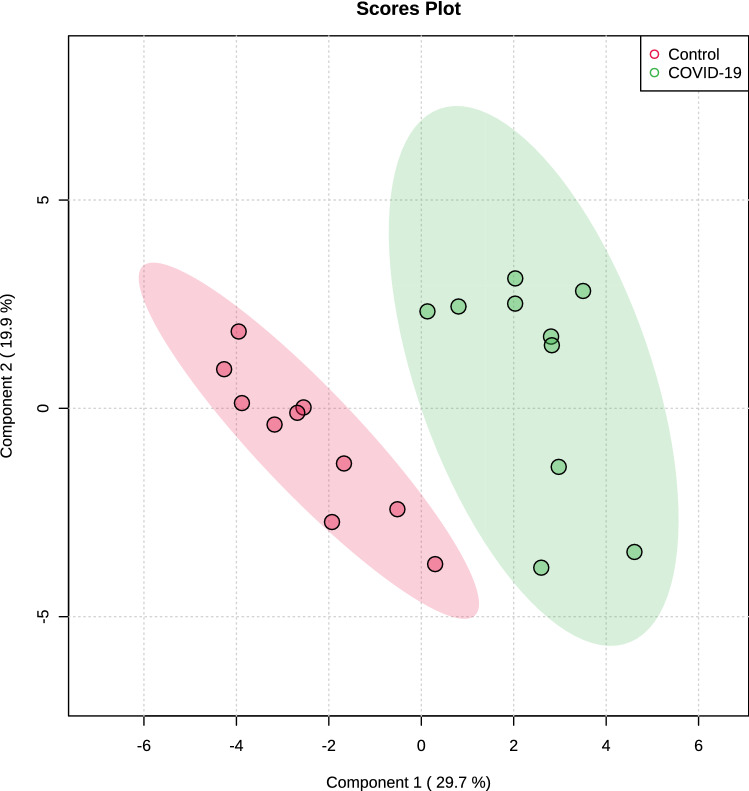
Partial least square-discriminant analysis (PLSD-DA), showing a robust separation among the groups (COVID-19 versus Control). Each point in the plot corresponds to a saliva sample. Source: Metaboanalyst 5.0 (https://www.metaboanalyst.ca).

**Figure 2:**
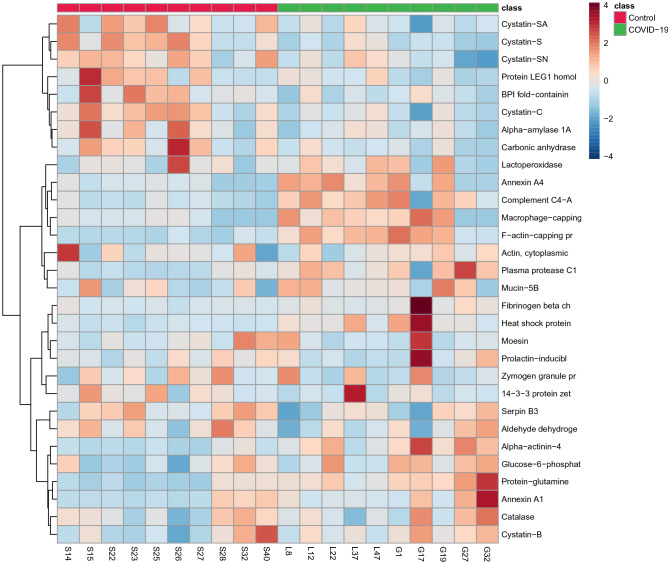
Hierarchical clustering differentiation of the 30 discriminative proteins in COVID-19 and control patients. Horizontal columns: relative abundance of each biologically relevant protein displaying distinct metabolic patterns between COVID-19 and control group. Each bar in the horizontal columns represents the relative abundance of the protein, where red bands indicate upregulated proteins and blue bands indicate downregulated proteins in the two groups. Source: Metaboanalyst 5.0 (https://www.metaboanalyst.ca).

**Figure 3:**
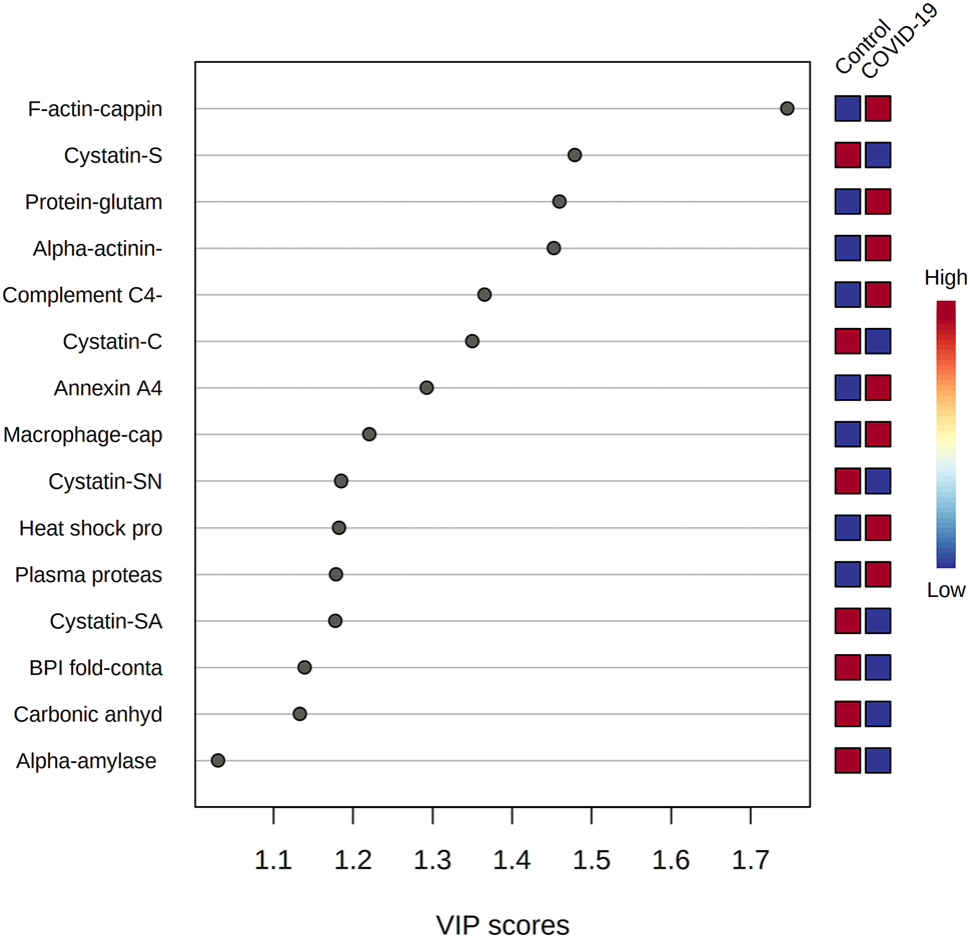
Variable Importance in Projection (VIP) represents the contribution score of the proteins that mostly contribute to the differentiation between COVID-19 and control group. The red and blue boxes on the right indicate the relative amounts of the corresponding protein in each group under study. Source: Metaboanalyst 5.0 (https://www.metaboanalyst.ca).

The multivariate analysis showed a robust separation between the COVID-19 group versus control in the partial least squares discriminant analysis (PLSDA) (accuracy = 0.85, R2 = 0.77, and Q2 = 0.73) (Fig. 1), which was also observed in the hierarchical cluster analysis (HCA) (Fig. 2). Furthermore, to differentiate the proteins that presented the biggest influence in the model, only proteins that showed a variable important projection (VIP) value greater than 1 were considered. Then, a total of 15 proteins were identified. Among them, eight proteins were upregulated and seven were downregulated in the saliva of patients with COVID-19 compared with controls. The upregulated proteins were: F-actin-capping protein subunit alpha-1 (CAZA1), alpha-actinin-4 (ACTN4), protein-glutamine gamma-glutamyltransferase (TGM3), complement C4-A (CO4A), annexin A4 (ANXA4), macrophage-capping protein (CAPG), heat shock protein HSP 90-beta (HSP90AB1) and plasma protease C1 inhibitor (SERPING1). The downregulated proteins were: cystatin-S (CST4), BPI fold-containing family B member 2 (BPIFB2), cystatin-C (CST3), cystatin-SN (CST1), cystatin-SA (CST2), and carbonic anhydrase 6 (CA6) (Fig. 3).

**Figure 4:**
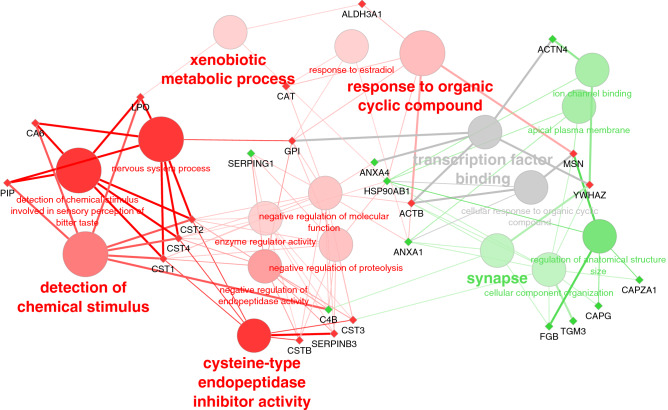
Interactome of GO terms differentially expressed in the saliva of COVID-19 patients, and their intermediate proteins. This analyse have been done with the Cytoscape application ClueGO and the REVIGO tool for GO terms selection. GO terms and proteins over-expressed in COVID-19 are in green, lower-expressed in COVID-19 are in red. GO terms in grey could not be attributed specifically to over or lower expressed terms/proteins. GO terms in bold represent GO terms selected to be the most representative of their GO group defined by the REVIGO tool. Source: ClueGO (v2.5.8) app for Cytoscape (v3.9.0).

Gene ontology (GO) enrichment analysis shows the most significantly enriched biological functions of up and down-regulated proteins in COVID-19 compared to control patients (Table 1). Due to the presence of a cluster of cystatins within the underexpressed proteins, cysteine-type endopeptidase activity (the molecular function of cystatins) and detection of chemical stimulus are clearly enriched, but also other GO terms like xenobiotic metabolic process, response to estradiol, and response to the organic cyclic compound. On the other hand, upregulated proteins were functionally more diverse and only a few GO terms were significantly enriched among them: synapse, ion channel binding, and regulation of anatomical structure (Fig. 4).

**Table 1:** Overrepresented GO terms within differentially expressed saliva proteins in COVID-19 patients.

Ontology	GO Accession	Name	*P*-value	Nr. Genes
Cellular Component	GO:0,045,202	Synapse	1.71E-02	5
	GO:0,016,324	Apical plasma membrane	1.99E-02	3
Biological Process	GO:0,009,593	Detection of chemical stimulus	1.68E-03	7
	GO:0,001,580	Detection of chemical stimulus involved in sensory perception of bitter taste	1.73E-03	6
	GO:0,050,877	Nervous system process	4.48E-03	7
	GO:0,014,070	Response to organic cyclic compound	4.48E-03	7
	GO:0,016,043	Cellular component organization	5.04E-03	10
	GO:0,004,869	Cysteine-type endopeptidase inhibitor activity	5.53E-03	6
	GO:0,090,066	Regulation of anatomical structure size	6.12E-03	5
	GO:0,045,861	Negative regulation of proteolysis	1.17E-02	10
	GO:0,044,092	Negative regulation of molecular function	1.46E-02	13
	GO:0,006,805	Xenobiotic metabolic process	1.99E-02	3
	GO:0,032,355	Response to estradiol	1.99E-02	3
	GO:0,030,234	Enzyme regulator activity	4.00E-02	9
	GO:0,010,951	Negative regulation of endopeptidase activity	4.00E-02	9
	GO:0,071,407	Cellular response to organic cyclic compound	4.91E-02	4
Molecular Function	GO:0,008,134	Transcription factor binding	6.12E-03	5
	GO:0,044,325	Ion channel binding	1.99E-02	3

### Validation of the proteomic results for total esterase activity and gamma-glutamyl transferase

**Figure 5:**
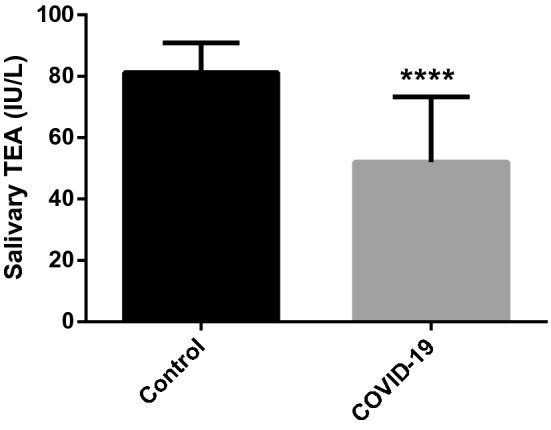
Total esterase activity concentrations in the saliva of COVID-19 patients (n = 66) and control group (n = 32). Asterisk indicates a significant difference between groups: ***p < 0.001. TEA: total esterase activity. Source: Prism 9.3, GraphPad software (https://www.graphpad.com).

The salivary total esterase activity (TEA) was significantly lower in patients with COVID-19 (mean±SD, 52.03 ± 21.28 IU/L) compared to healthy individuals (81.23 ± 9.67 IU/L) (P<0.001) (Fig. 5).

The analytical validation of the gamma glutamyl-transferase (gGT) automated assay showed an intra- and inter-assay imprecision less than 10% and high linearity (R > 0.99) to the measure of salivary gGT after serial dilutions of a saliva sample with high concentration. The low limit of quantification (LLOQ) and limit of detection (LoD) were set at 4.24 and 2.95 IU/L, respectively.

**Figure 6:**
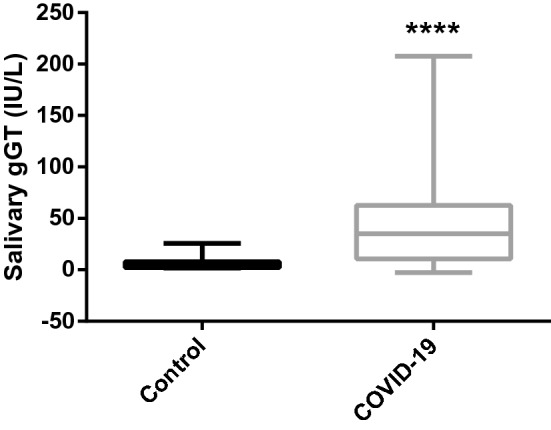
Salivary gamma glutamyl-transferase concentrations in the saliva of COVID-19 patients (n = 66) and control group (n = 32). The plot shows the median (line within box), 25th and 75th percentiles (box), and 10th and 90th percentiles (whiskers). Asterisk indicates a significant difference between groups: ***p < 0.001. gGT: gamma glutamyl-transferase. Source: Prism 9.3, GraphPad software (https://www.graphpad.com).

The activities of gGT were significantly higher in patients with COVID-19 (median and range: 35.2, 10.6–207.5 IU/L) compared to healthy individuals (4.1 (1.5–25.5) IU/L) (Fig. 6).

### Differences in the salivary protein profile in individuals with different COVID-19 disease severity

 The comparison of mild disease COVID-19 patients versus controls showed that ten proteins were differentially abundant, being four downregulated and six upregulated (Table 2). In the case of comparison between severe disease COVID-19 patients and controls, 15 proteins were differentially abundant, being 13 downregulated and two upregulated (Table 3).

**Table 2:** Differentially expressed proteins in mild COVID-19 (n = 5) compared with healthy individuals (n = 10).

Accession (UniProt)	Protein name	Max. no. of quantified peptides	Molecular function	K-W test (P-value)	Dunn’ test for multiple comparison		Regulation in COVID-19 mild disease
					Log2 fold change	*P*-value	
P30838	Aldehyde dehydrogenase, dimeric NADP-preferring	2	3-chloroallyl aldehyde dehydrogenase activity	0.01*	−1.29	0.006**	Down
Q8N4F0	BPI fold-containing family B member 2	5	Lipid binding	0.003**	−1.19	0.01*	Down
P29508	Serpin B3	5	Serine-type endopeptidase inhibitor activity	0.01*	−1.08	0.01*	Down
P12273	Prolactin-inducible protein	2	Actin binding	0.04*	−0.72	0.01*	Down
P04792	Heat shock protein beta-1	2	Protein kinase C binding	0.04*	1.21	0.04*	Up
P09525	Annexin A4	3	Calcium ion binding	0.01*	1.00	0.006**	Up
Q13510	Acid ceramidase	2	Hydrolase activity	0.02*	0.90	0.01*	Up
P52907	F-actin-capping protein subunit alpha-1	2	Actin binding	0.04*	0.79	0.01*	Up
P01619	Immunoglobulin kappa variable 3–20	2	Antigen binding	0.02*	0.72	0.009**	Up
P0C0L4	Complement C4-A	6	Endopeptidase inhibitor activity	0.04*	0.71	0.01*	Up

K-W: Kruskal–Wallis.

**Table 3:** Differentially expressed proteins in severe COVID-19 (n = 5) compared with healthy individuals (n = 10).

Accession (UniProt)	Protein name	Max. no. of quantified peptides	Molecular function	K-W test (*P*-value)	Dunn’ test for multiple comparison		Regulation in COVID-19 mild disease
					Log2 fold change	*P*-value	
P01036	Cystatin-S	6	Cystein-type endopetidase inhibitor activity	0.002**	−2.54	0.001**	Down
Q6P5S2	Protein LEG1 homolog	5		0.02*	−2.32	0.006**	Down
P01034	Cystatin-C	2	Peptidase inhibitor activity	0.005**	−1.78	0.002**	Down
P23280	Carbonic anhydrase 6	5	Zinc ion binding	0.01*	−1.78	0.007**	Down
P09228	Cystatin-SA	5	Cystein-type endopetidase inhibitor activity	0.008**	−1.61	0.002**	Down
P01037	Cystatin-SN	5	Cystein-type endopetidase inhibitor activity	0.004**	−1.57	0.001**	Down
Q8N4F0	BPI fold-containing family B member 2	5	Lipid binding	0.003**	−1.43	0.003**	Down
P0DUB6	Alpha-amylase 1A	3	Calcium ion binding	0.009**	−1.40	0.003**	Down
P29401	Transketolase	4	Calcium ion binding	0.04*	−1.37	0.02*	Down
Q96DA0	Zymogen granule protein 16 homolog B	3	Carbohydrate binding	0.04*	−1.05	0.01*	Down
P29508	Serpin B3	5	Serine-type endopeptidase inhibitor activity	0.01*	−1.00	0.02*	Down
P26038	Moesin	4	Cell adhesion molecule binding	0.02*	−0.85	0.008**	Down
P60709	Actin cytoplasmatic 1	6	ATP binding	0.03*	−0.68	0.01*	Down
P02675	Fibrinogen beta chain	5	Structural molecule activity	0.04*	1.02	0.01*	Up
Q43707	Apha-actinin-4	10	Calcium ion binding	0.01*	0.73	0.005**	Up

K-W: Kruskal–Wallis.

**Figure 7:**
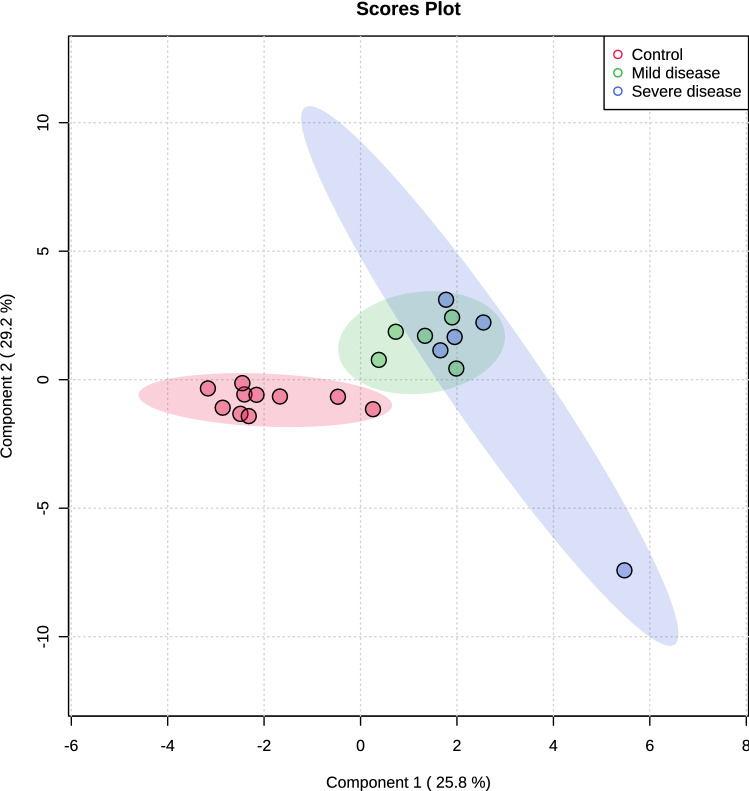
Partial least square-discriminant analysis (PLSD-DA) in patients with different COVID-19 severity and controls. Each point in the plot corresponds to a saliva sample. Source: Metaboanalyst 5.0 (https://www.metaboanalyst.ca).

**Figure 8:**
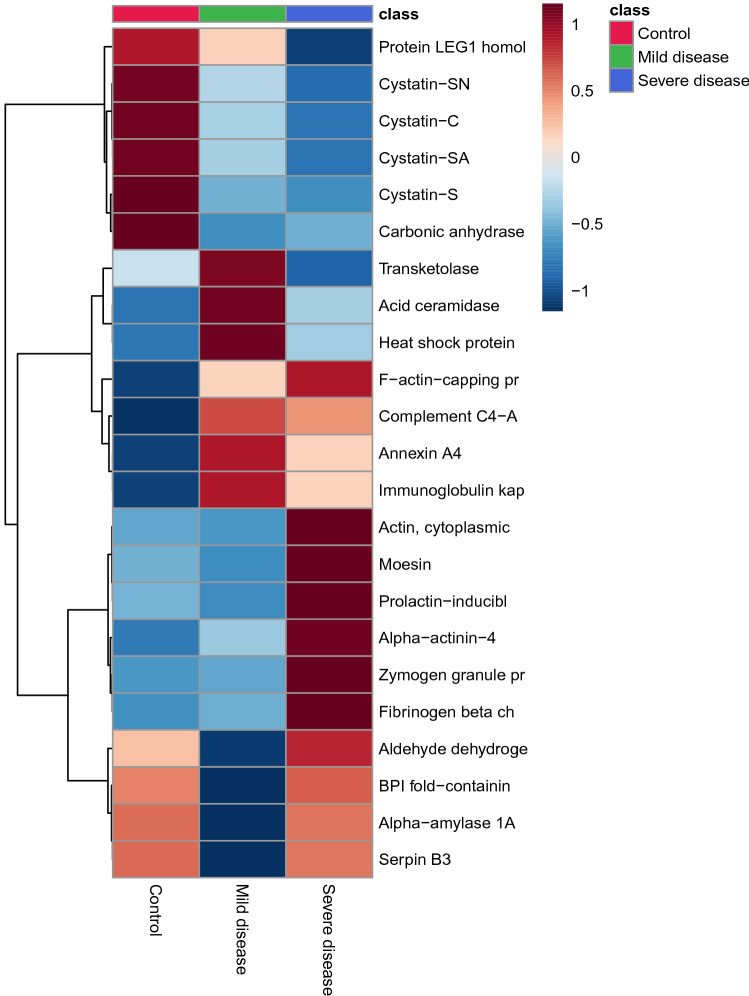
Hierarchical clustering differentiation of the top significant proteins in COVID-19 patients with different severity and controls. Horizontal columns: mean group expression of each biologically relevant protein displaying distinct metabolic patterns between the different severity COVID-19 and control patients. Each bar in the horizontal columns represents the mean expression, where red bands indicate upregulated metabolites and blue bands indicate downregulated proteins within the three groups. Source: Metaboanalyst 5.0 (https://www.metaboanalyst.ca).

**Figure 9:**
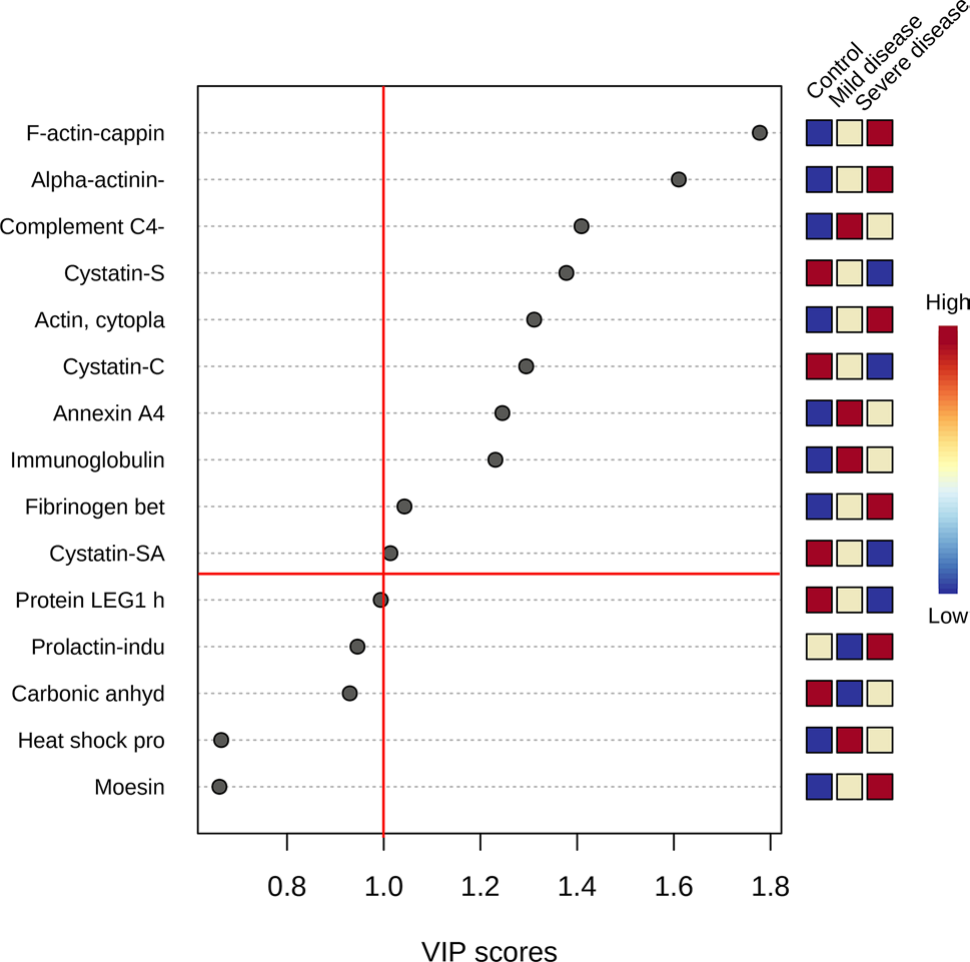
Variable Importance in Projection (VIP) represents the contribution score of the proteins that mostly contribute to the differentiation within the different COVID-19 severities and the control group. The red and blue boxes on the right indicate the relative amounts of the corresponding protein in each group under study. Horizontal and vertical red lines are delimiting the proteins with VIP value above 1. Source: Metaboanalyst 5.0 (https://www.metaboanalyst.ca).

Multivariate analysis showed an overlapping in the COVID-19 patients when grouping by severity with the moderate ability of the model for prediction (accuracy = 0.80, R2 = 0.51, and Q2 = 0.53) (Fig. 7). The HCA highlighted the protein cluster separation between groups (Fig. 8). After partial least squares discriminant analysis (PLS-DA), ten proteins showed the highest differentiation between the different COVID-19 severities when compared with controls, showing a VIP value greater than 1 (Fig. 9). Among them, six proteins were upregulated and four were downregulated in mild or severe COVID 19 disease compared with healthy individuals. Specifically, within upregulated, CAZA1, CO4A, ANXA4, and immunoglobulin kappa variable 3–20 (IGKV3-20) were significantly increased in mild disease COVID-19 while ACTN4 and fibrinogen beta (FGB) chain were significantly increased in severe COVID-19 individuals. In the case of downregulated proteins, the cystatins CST2, CST3, CST4, and actin cytoplasmatic 1 (ACTB) were significantly decreased in severe disease. No one showed a significant decrease in mild disease patients. Additionally, two proteins changed when comparing mild versus severe COVID-19 patients. These proteins were found downregulated and they were ANXA4 (log2(FC): -0.96, p-value: 0.04) and IGKV3-20 (log2(FC): -0.86, p-value: 0.02).

**Figure 10:**
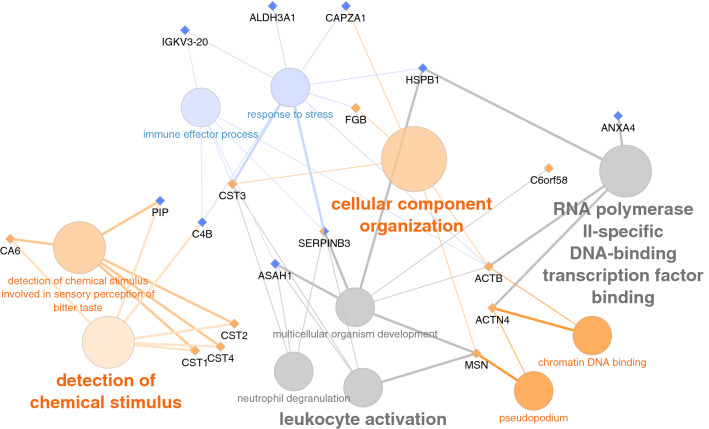
Interactome of GO terms differentially expressed in the saliva of COVID-19 patients depending of disease severity, and their intermediate proteins. This analyses have been done with the Cytoscape application ClueGO and the REVIGO tool for GO terms selection. GO terms and proteins related to mild COVID-19 are in blue, and those related to severe COVID-19 are in orange. GO terms in grey could not be attributed specifically to any disease severity. Source: ClueGO (v2.5.8) app for Cytoscape (v3.9.0).

GO terms altered in patients with mild COVID-19 were a response to stress and immune effector process while those altered in severe COVID-19 patients were related to the detection of chemical stimulus, cellular component organization, pseudopodium, and chromatin DNA binding (Fig. 10).

## Discussion

In this preliminary study, we describe, for the first time, the salivary proteome variations that can be observed in patients with  COVID-19. The study of alterations in the plasma proteome of COVID-19 patients has been recently explored with the identification of some proteins that may act as promising biomarkers for the disease physiopathology^15^. Saliva is easier to collect than serum and can be a source of biomarkers for clinical use in COVID-19^4^.

The high-resolution proteomics analysis performed in this work identified a different pattern in salivary proteins in patients with COVID-19 when compared with healthy individuals. Within the salivary proteins that were significantly increased in patients with COVID-19, five contributed most on the model: CAZA1, ACTN4, TGM3, CO4A, and ANXA4.

Two of these upregulated proteins are actin-binding proteins (CAZA1 and ACTN4) that could be related to an airway defense capability, while ANXA4 seems to present anticoagulant properties. CAZA1 has been identified as a protein closely related to the damage-associate molecular pattern (DAMP) high-mobility group box-1 (HMGB1), that is involved in the regulation of epithelial cellular and immune homeostasis of airway epithelial cells^16^. DAMPs are responsible for the liberation of local immune cells to elicit the activation of the innate and adaptive immune response^17^. The increased levels of CAZA1 in the saliva of COVID-19 individuals could be related to a protective role that tries to reduce the impairments in the airway mucosal immunity during this disease. The protecting role of CAZA1 has been proven in a study in which its deficiency has been shown to facilitate the invasion as well as metastasis of liver cancer cells^18^. ACTN4 is a non-muscle isoform belonging to the α-actinins (ACTNs) family that are cytoskeletal proteins that maintain cytoskeleton integrity and control cell movement^19^. ﻿The downregulation of ACTN4 expression contributes to endothelial injury by promoting apoptosis^20^ and the finding in our study of being upregulated in the saliva of patients with COVID-19 could be related to a protective function. Finally, we found upregulated the ANXA4. Annexins (ANXs) are a family of multifunctional Ca2 + -binding proteins that bind to anionic biomolecules^21^. One well-known physiological role of several ANXs is the regulation of blood coagulability during coagulation (including thrombosis) and fibrinolysis^22^. It has been suggested that serum ANXA4 reduces prothrombotic events in different conditions and may act as an anticoagulant protein in specific situations such as pregnancy^23^. Additionally, ANXA4 is able to inhibit the intrinsic pathway of coagulation by blocking the auto-activation of coagulation factor XII^24^. Therefore, its upregulation in COVID-19 could be a compensatory effort to prevent coagulability disorders.

TGM3 is a member of the transglutaminases family which are enzymes that catalyze the crosslinking of a glutaminyl residue of a protein/peptide substrate to a lysyl-residue of a protein/peptide co-substrate. The importance of transglutaminases in viral infections has been pointed out since they cause modification of surface viral glycoproteins gp41 and gp120 that mediate penetration of HIV into the cell^25^. TGM3 has been linked to the normal epithelial function in oral cavity^26^. In addition, the elevation of this enzyme has been related to oxidative damage of pulmonary tissue^27^. Further studies in a large population of patients with COVID-19 would be needed to elucidate if TGM3 could be related to the ability of SARS-CoV-2 to infect epithelial airway cells and act as a biomarker for pulmonary damage associated with COVID-19. The increase in the activity of gGT found in our study could reflect the increase in the salivary levels of TGM3 in patients infected by SARS-CoV-2. However in addition of TGM3, other TGMs can have influence on the gGT activity and, therefore, it should be pointed out that the measurement of gGT is less specific than the direct quantification of TGM3.

CO4A is considered an essential component in the complement system; it plays an important role in the activation of classical and lectin complement cascades^28^. The upregulation of CO4A found in the saliva of patients with COVID-19 infection might cause endothelial disruption, as previously postulated^28^ being one of the mechanisms to induce microvascular thrombi in these patients.

The most important downregulated proteins were the cystatins type 2 family 1 (CST1), 2 (CST2), 3 (CST3), and 4 (CST4), and carbonic anhydrase (CA6). As the GO analysis clearly highlights, these proteins are involved in the detection of chemical stimulus in the perception of bitter taste, thus suggesting this process could be impaired or dysregulated in COVID-19 patients. In fact, it has been shown previously that salivary cystatins can modulate the bitter taste in adults^29^ and infants^30^. This finding is in line with the well-known perturbations in sensory perception caused by COVID-19, which can result in the complete loss (anosmia, ageusia) but also alterations (dysgeusia) of the senses of taste and smell^31,32^ and could help interpreting the molecular mechanisms behind these processes. Indeed, CA6 deficiency has been shown to cause abnormal bitter taste perception in a mouse model^33^. Moreover, the expression of CA6 is considered essential for maintaining homeostasis on the surfaces of the oral cavity and upper alimentary canal^32^. Therefore, the reduction in its expression levels showed in our study could be implicated in the dysregulation of local airway homeostasis caused by SARS-CoV-2 infection. CA6 is a zinc-containing metalloenzyme able to catalyze the reversible conversion of carbon dioxide and water into bicarbonate an proton, giving an important role in pH regulation^34^. In a previous report, it has been demonstrated that CA6 is one of the major contributors to TEA using 4-NA as substrate for its measurement^35^. Therefore, we used the measurement of a total esterase by a spectrophotometric assay with 4-NA as substrate to validate the CA6 proteomics results in a larger population of patients, in which decreases in TEA were found, thus further supporting the potential use of CA6 as a biomarker.

Upregulation of gGT and the downregulation of TEA in the saliva of patients with SARS-CoV-19, would reflect an upregulation of TGM3 and a downregulation of CA6, respectively, and therefore could be related to the presence of disturbances at the respiratory tract level. This could give to these biomarkers the potential to evaluate the presence of respiratory complications, since increases in gGT and decreases in TEA could indicate an involvement of the respiratory tract in a patient with COVID-19. Further studies with a large population of affected individuals should be made to test these potential applications.

In our study, there was an upregulation in FGB and a downregulation in cystatins in patients with severe COVID-19 compare with those that had mild COVID-19. The presence of salivary fibrinogen gamma chain (FGG) promotes viral aggregation and activation in cases of vesicular stomatitis virus (VSV) infection^36^. Therefore, the increment of FGB detected in severe patients could favor viral aggregation and activation. If this hypothesis is confirmed, salivary FGB could be a marker of severity in COVID-19. Further studies should be performed to confirm this hypothesis. Similarly, cystatins were found decreased in severe cases compared with mild cases, denoting that the changes associated with these proteins related to the loss of taste and the affectation of the nervous system are more representative in patients with severe disease.

This study presents some limitations and the results should be taken with caution. First, the relatively small number of samples used in the proteomic trial may have an impact on the results. Although two of the proteins that showed significant changes were validated, ideally other proteins showing significant changes in the proteomic study should have been validated in a large population. In our study, for the validation of the proteomic results, two proteins that could be easily measured by automated assays and feasible to apply in routine analysis were selected, however, ideally, the potential biomarkers identified by proteomic analysis should be validated using immunochemical assays. Also, only men were used in the proteomic study and although no differences between healthy and diseased individuals were found when the data of the assays of gGT and TEA were compared between males and females, possible differences in gender in other proteins should be explored in the future. In addition, some changes in the salivary proteome appeared in COVID-19 affected individuals could be similar to other viral infections and therefore further comparison using individuals affected with other viral infections would be necessary to determine which protein changes are specific of COVID-19. Although we found a good prediction model for the differentiation between COVID-19 and control patients, the ability of the model in differentiating between disease severities was moderate and, therefore, further investigations should be carried out to confirm the changes and the importance of the differentially observed markers.

## Conclusion

Our results showed that there are changes in the salivary proteome of COVID-19 patients. These changes could be related to the protective response to viral infection and the altered sensory taste perception that occurs during the disease. Moreover, there are two proteins, gGT, and TEA, which have shown potential as biomarkers of respiratory disturbances occurring during COVID-19.

## Material and methods

### Study design and subjects

- Proteomic study: This case–control study consisted of two different groups: (1) COVID group, composed of ten confirmed positive COVID-19 cases, including five men (aged between 35 and 64 years old) with mild infection (no need for oxygen supplementation or conventional oxygen therapy) and five men (aged between 21 and 67 years old) with severe infection (requiring nasal flow oxygen or assisted respiration)^37^; and (2) control group, based on ten healthy men (aged between 28 and 55 years old) who did not show any sign of disease for at least four weeks, and tested negative for COVID-19 in real time-polymerase chain reaction (RT-PCR).
- Validation study: The validation study employed 98 additional saliva samples separated into two groups: (1) COVID-19 group, integrated by 66 patients tested positive for COVID-19 (34 males and 32 females aged between 21 and 91 years old), and (2) control group, composed of 32 healthy individuals (16 men and 16 women aged between 24 and 50 years old) who did not show any sign of disease for at least four weeks, and were negative for COVID-19 in RT-PCR.

All patients were admitted to “Reina Sofia” hospital of Murcia (Spain) between September and December 2020. COVID-19 was confirmed by ribonucleic acid (RNA) extraction and quantification by RT-PCR with a commercial kit (FTD SARS-CoV-2, Siemens) from nasopharyngeal swabs (NPS). ﻿Samples from healthy individuals included in both studies were taken from volunteer participants who belonged to the staff of the University of Murcia.

The study was in accordance with the ethical standards of the Institutional and/or National Research Committee (1349/2016) and with the 1964 Helsinki Declaration and its later amendments or comparable ethical standards. All participants were informed of the purpose and experimental procedures of the study and signed a written informed consent form before their participation. This study was approved by the Ethical Committee of the University of Murcia and the Ethical Committee of the IMIB-Arrixaca.

### Saliva collection

﻿Saliva samples were taken under supervision using the drainage technique in 5 mL polystyrene tubes with a collection time of 5 min. The samples were collected at about the same time in all subjects (8:00–11:00 a.m.). Immediately after collection, samples were placed in coolers and transported to the laboratory, where saliva was vortexed and centrifuged (3000 × g for 10 min at 4 °C), and the supernatant was transferred into polypropylene tubes and refrigerated until the inactivation procedure. No samples showed blood contamination as determined by visual inspection.

### Biosafety and pathogen inactivation

﻿A chemical non-ionic inactivation protocol with NP-40 was performed in all salivary samples prior to TMT procedures to eliminate the infectious capability of the SARS-CoV-2 virus^38^. In brief, ﻿inactivation was performed by adding NP-40 (10% v/v) to a final 0.5% NP-40 concentration followed by 30 min of incubation at room temperature. The inactivated saliva samples were stored at the Biobank of the Imib Center (Murcia, Spain) at − 80 °C until proteomics analysis.

### Liquid chromatography-tandem mass spectrometry (LC/MS–MS)

﻿From each sample, 35 µg of acetone-precipitated proteins were subjected to reduction, alkylation, and digestion and were labeled using 10-plex TMT reagents (Thermo Scientific, Rockford, IL, USA) according to manufacturer instructions (Thermo Scientific, Waltham, MA, USA). Total protein concentration of salivary samples was determined using bicinchoninic acid (BCA) assay (Thermo Scientific, Rockford, USA). The internal standard used in the TMT 10-plex experiments was a pooled sample with equal protein amounts of all 20 samples. Samples were processed as previously described^39^ and stored at − 80 °C before further liquid chromatography-tandem mass spectrometry (LC–MS/MS) analysis.

The LC–MS/MS analysis was performed by using an Ultimate 3000 RSLCnano flow system (Dionex, Germering, Germany)) coupled to a Q Exactive Plus mass spectrometer (Thermo Fisher Scientific, Bremen, Germany) as described previously^40^. Peptides were dissolved in loading solvent (1% ACN, 0.1% formic acid) and loaded onto the trap column (C18 PepMap100, 5 μm, 100A, 300 μm × 5 mm), desalted for 12 min at the flow rate of 15 μL/min and separated on the analytical column (PepMap™ RSLC C18, 50 cm × 75 μm) using a linear gradient of 5–45% mobile phase B (0.1% formic acid in 80% ACN) over 120 min, 45% to 90% for 2 min, held at 80% for 2 min and re-equilibrated at 5% B for 20 min at the flow rate of 300 nL/min. Mobile phase A consisted of 0.1% formic acid in water. Ionisation was achieved using nanospray Flex ion source (Thermo Fisher Scientific, Bremen, Germany) containing a 10 μm-inner diameter SilicaTip emitter (New Objective, Woburn, MA, USA). The MS operated in positive ion mode using DDA Top8 method. Full scan MS spectra were acquired in range from m/z 350.0 to m/z 1800.0 with a resolution of 70,000, 110 ms injection time, AGC target 1E6, a ± 2.0 Da isolation window and the dynamic exclusion 30 s. HCD fragmentation was performed at step collision energy (29% and 35% NCE) with a resolution of 17,500 and AGC target of 2E5.

For protein identification, a database search against Homo sapiens FASTA files (downloaded from Uniprot database on April 30th, 2019, 262,540 sequences) was performed in Proteome Discoverer (version 2.3., Thermo Fisher Scientific). For reporting confidently identified proteins, at least two unique peptides and 5% FDR were required. Protein quantification was achieved by correlating the relative intensities of reporter ions extracted from the tandem mass spectrum to that of the peptides selected for MS/MS fragmentation. For comparison of relative quantification results between the experiments, the internal standard was used.

### Measurement of total esterase activity (TEA) and gamma glutamyl-transferase (gGT) assays for the validation study

TEA was measured by using an automated assay previously validated for use in human saliva^35^.

A spectrophotometric assay for gGT assay that was non-previously validated for use in human saliva was analytically validated based on the following parameters:

- Precision: The intra- and inter-assay coefficient of variation (CV) were calculated after analyzing two samples of different concentrations (one sample with high concentration and one sample with low concentration) five times in a single assay run and five times in five consecutive days, respectively.
- Accuracy was indirectly evaluated by linearity under dilution. Thus, one saliva sample with a high concentration was serially diluted with saline solution.
- LOD was defined as the lowest analyte concentration that could be distinguished from a specimen of zero value. It was calculated based on data from ten replicate measurements of the zero standard (saline solution) as mean value plus three standard deviations (SD).
- LLOQ was calculated based on the lowest analyte concentration that could be measured with an intra-assay < 20%.

### Statistical analysis

#### Bioinformatics and proteomic data analysis

﻿Fold changes between groups were calculated as the log2(Mean(Group2)/Mean(Group1)). Due to the small number of individuals, normal distribution of values was not assumed and thus non-parametric tests were used to assess the statistical significance of differentially expressed proteins between groups. For univariate analysis, the Mann–Whitney U rank test was used when comparing the COVID-19 group with the control group. Kruskal–Wallis H-test was used to search for significant differences in protein expression between the two different COVID-19 severities and control groups. For those proteins, Dunn’s multiple comparison test was used in a posthoc analysis to differentiate the specific variation between these groups. Statistical significance was considered when *P* < 0.05. All statistical analyses were implemented using Python3 and the SciPy^41^ and scikit-posthoc^42^ libraries. The determined differentially expressed protein datasets were subjected to multivariate analysis, including PLS-DA and HCA using MetaboAnalyst 4.0 to highlight the discrimination between the experimental data. Proteins with VIP values above 1.0 were considered the most discriminant in the model. For the multivariate analysis data was normalized by a pooled sample from the control group, and autoscaling was used.

Proteins were mapped to UniProt^43^ entries and then annotated with gene names, protein names, and descriptions. For functional characterization of the differentially expressed proteins, GO enrichment analysis was performed using the Cytoscape plugin ClueGo^44^ and its functionalities to fuse and group functionally related terms to reduce redundancy.

#### Validation data analysis

The analysis of data for the validation of gGT showed non-parametric distribution and therefore, the Mann–Whitney test was employed to test the significance between groups. Results were expressed as median and range. The data of TEA showed a parametric distribution and was analyzed using a t-test, expressing the results as mean and SD. All data included in the validation study were analyzed using GraphPad Prism software for Windows 10.

The statistical power was determined using the values obtained in each validated protein to verify the null hypothesis. By using the mean and standard deviation of TEA and gGT concentrations in healthy and COVID-19 patients and a power of 80% at a 5% level of significance, the number of individuals was calculated using the G-Power software y^45^. The power analysis test indicated that ten subjects (five for the healthy and five for the COVID-19 group) were required to obtain a power of 80% with a 5% level of significance in the case of TEA and 20 subjects (ten for the healthy and ten for the COVID-19 group) for gGT validation.

## Supplementary Information

Supplementary Information.

## Data Availability

Proteomic data is available via ProteomeXchange with identifier PXD031318.
