# Supplementary material for: Saliva changes in composition associated to COVID-19: a preliminary study
Source: Sci Rep. 2022 Jun 27;12:10879. doi: 10.1038/s41598-022-14830-6 (PMC9237082; doi:10.1038/s41598-022-14830-6)
Supplement: Supplementary file 1 — Supplementary Information. [file 41598_2022_14830_MOESM1_ESM.docx]

**Supplementary material**

**Supplementary Table S1.** Differentially expressed proteins between COVID-19 (n=10) and control group (n=10).

| Accession (UniProt) | Protein name | Max. no. of quantified peptides | Molecular function | Log2(FC) | Mann-Whitney (P-value) | Regulation in COVID-19 |
| --- | --- | --- | --- | --- | --- | --- |
| Q8N4F0 | BPI fold-containing family B member 2 | 5 | Lipid binding | -1.3 | 0.0005*** | Down |
| P01036 | Cystatin-S | 6 | Cystein-type endopeptidase inhibitor activity | -1.84 | 0.0008*** | Down |
| P30838 | Cystatin-C | 2 | Peptidase inhibitor activity | -1.35 | 0.001** | Down |
| P01036 | Cystatin-SN | 5 | Cysteine-type endopeptidase inhibitor activity | -1.10 | 0.001** | Down |
| P29508 | Serpin B3 | 5 | Serine-type endopetidase inhibitor activity | -1.03 | 0.002** | Down |
| P0DUB6 | Alpha-amylase 1A | 3 | Calcium ion binding | -1.10 | 0.002** | Down |
| P23280 | Carbonic anhydrase 6 | 5 | Zinc ion binding | -1.45 | 0.002** | Down |
| P09228 | Cystatin-SA | 5 | Cystein-type endopeptidase inhibitor activity | -1.18 | 0.002** | Down |
| P30838 | Aldehyde dehydrogenase, dimeric NADP-preferring | 2 | 3-chloroallyl aldehyde dehydrogenase activity | -0.94 | 0.004** | Down |
| P60709 | Actin, cytoplasmic 1 | 6 | ATP binding | -0.58 | 0.007** | Down |
| P26038 | Moesin | 4 | Cell adhesion molecule binding | -0.64 | 0.007** | Down |
| Q96DA0 | Zymogen granule protein 16 homolog B | 3 | Carbohydrate binding | -0.65 | 0.01* | Down |
| P12273 | Prolactin-inducible protein | 2 | Actin binding | -0.53 | 0.01* | Down |
| P63104 | 14-3-3 protein zeta/delta | 2 | Protein kinase binding | -0.69 | 0.01* | Down |
| Q9HC84 | Mucin-5B | 20 |  | -0.37 | 0.02* | Down |
| Q6P5S2 | Protein LEG1 homolog | 5 |  | -1.4 | 0.02* | Down |
| P04040 | Catalase | 9 | Heme binding | -0.43 | 0.03* | Down |
| P06744 | Glucose-6-phosphate isomerase | 4 | Cytokine activity | -0.29 | 0.03* | Down |
| P22079 | Lactoperoxidase | 10 | Metal ion binding | -0.48 | 0.04* | Down |
| P04080 | Cystatin-B | 2 | Cystein-type endopeptidase inhibitor activity | -0.29 | 0.04* | Down |
| Q43707 | Alpha-actinin-4 | 10 | Nuclear receptor coactivator activity | 0.57 | 0.004** | Up |
| P05155 | Plasma protease C1 inhibitor | 2 | Serine-type endopetidase inhibitor activity | 0.30 | 0.01* | Up |
| Q08188 | Protein-glutamine gamma-glutamyl transferase E | 8 | Protein-glutamine gamma-glutamyl transferase activity | 0.43 | 0.02* | Up |
| P02675 | Fibrinogen beta chain | 5 | Structural molecule activity | 0.67 | 0.02* | Up |
| P08238 | Heat shock protein HSP 90-beta | 2 | Heat shock protein binding | 0.80 | 0.02* | Up |
| P04083 | Annexin A1 | 8 | Double-stranded DNA helicase activity | 0.64 | 0.03* | Up |
| P09525 | Annexin A4 | 3 | Calcium ion binding | 0.60 | 0.03* | Up |
| P40121 | Macrophage-capping protein | 2 | Protein-containing complex binding | 0.66 | 0.03* | Up |
| P0C0L4 | Complement C4-A | 6 | Endopeptidase inhibitor activity | 0.66 | 0.03* | Up |
| P52907 | F-actin-capping protein subunit alpha-1 | 2 | Actin binding | 0.57 | 0.04* | Up |

FC: fold change; COVID-19: coronavirus disease 2019; *P-value <0.05; **P-value<0.01; ***P-value<0.001.
